# An attempt to evaluate STEAM project-based instruction from a school mathematics perspective

**DOI:** 10.1007/s11858-021-01303-9

**Published:** 2021-09-03

**Authors:** Jose-Manuel Diego-Mantecon, Theodosia Prodromou, Zsolt Lavicza, Teresa F. Blanco, Zaira Ortiz-Laso

**Affiliations:** 1 University of Cantabria, Santander, Spain; 2 University of New England, Armidale, Australia; 3 Johannes Kepler University, Linz, Austria; 4 University of Santiago de Compostela, Santiago de Compostela, Spain

**Keywords:** Mathematics learning, STEM education, STEAM education, Project-based learning, Classroom implementation

## Abstract

Official documents in several educational systems reflect the importance of integrating Science, Technology, Engineering, Arts, and Mathematics (STEAM) and consider project-based learning (PBL) as a way of integrating such disciplines in the classroom. Although STEAM-PBL has been characterized and evaluated in different ways, its impact on school mathematics teaching remains unclear. Mathematics is recognized as the fundamental basis of other disciplines; however, many students still perceive it as a difficult subject and abandon it. To analyze STEAM-PBL classroom implementation from a school mathematics standpoint, we examined 41 classroom experiences from 11 Spanish secondary education teachers (five in-field mathematics teachers), who participated in a STEAM training program for more than 4 years. To frame this study, Thibaut et al.’s (J STEM Educ 3(1):02, 2018) and Schoenfeld’s (Educ Res 43(8):404–412, 2014) characterizations of well-designed and implemented projects, respectively, were employed. The results showed that in-field mathematics teachers avoided transdisciplinary projects in which school mathematics is difficult to address, while out-of-field teachers tended to overlook the mathematics in interdisciplinary projects. Unlike out-of-field teachers, mathematics teachers often eluded design-based learning processes for deeply exploiting school mathematics. The latter teachers promoted high cognitive demands and positive perceptions about mathematics in projects where formative environments were generated through discussion and a meaningful feedback loop.

## Introduction

We live in a society where Science, Technology, Engineering, and Mathematics (STEM) are fundamental disciplines. Mathematics is central to many professions, but it is perceived as difficult and many students leave it, closing doors to scientific, engineering, and technological careers (Li & Schoenfeld, 2019). The Organisation for Economic Co-operation and Development (OECD, 2019b) recently reported that 15-year-old students may not achieve the minimum level of mathematics competency. Educational systems worldwide have attempted to minimize the gap between the demands of the current society and academic training.

Real-life contexts and STEM workplaces demand knowledge and skills that extend beyond the four disciplines. Citizens would need not only to master content from various disciplines but also to solve ill-defined problems through reasoning, which involves interpreting real situations, making assumptions, devising strategies, and verifying solutions (OECD, 2019a). Currently, there exist a number of initiatives that raise optimism (Maass et al., 2019a). Such initiatives are characterised by a STEM focus (Maass et al., 2019a, b) and a project based-learning implementation (PBL, Diego-Mantecón et al., 2021). Several authors have emphasised the appropriateness of PBL for instructing STEAM education; the acronym emerged from incorporating the A of Arts in STEM (Colucci-Gray et al., 2019; Herro & Quigley, 2017). However, evaluations of STEM-PBL and STEAM-PBL (from now on STE(A)M-PBL) do not present conclusive results. Although benefits of this approach are described for promoting learning in general (e.g., attitudinal competence development, opportunities to deal with real problems, and collaborative learning), many authors, especially from the mathematics field, remain dubious about its potential to support mathematics learning (Diego-Mantecón et al., accepted; Godino et al., 2015; Lasa et al., 2020).

Some researchers suggest STE(A)M-PBL increases STEM marks of low-average performing students (Han et al., 2015, 2016), while others brought out that this approach offers minimal mathematical content—usually basic and utilitarian (Lasa et al., 2020)—which does not improve mathematics achievement but generates positive attitudes toward this discipline (Diego-Mantecón et al., 2019). Particularly, Godino et al. (2015) stated that this approach is characterised by a lack of teacher guidance which does not stimulate feedback, and thus learning, on single disciplines like mathematics. Regardless, the number of studies evaluating STE(A)M-PBL instruction from a mathematics learning perspective is rather low, which makes it difficult to make conclusions. Lavicza et al. (2020) and Li and Schoenfeld (2019) confirm that theoretical changes in mathematics education have fallen far short, not reaching classroom implementation at the expected level. To get insights about the impact of STE(A)M-PBL on mathematics learning, in this study we examine 41 instances of instruction by 11 Spanish teachers (five in-field mathematics teachers) participating in a STEAM professional-development program undertaken at the University of Cantabria since 2015. To carry out such an analysis, Thibaut et al.’s (2018) and Schoenfeld’s (2014) characterisations of well-designed and instructed STE(A)M projects, respectively, were applied.

## STE(A)M project-based learning

Researchers define STEM education as practices integrating content and skills from science, technology, engineering, and/or mathematics. These practices are usually framed in real world contexts promoting problem solving, inquiry-based, and collaborative learning (Martín-Páez et al., 2019; Thibaut et al., 2018). Promoters of educational trends advocate for incorporating the A of Arts, in the so-called STEAM approach. This approach is highly associated with creativity, ethics, aesthetic, and innovation (Colucci-Gray et al., 2019; Quigley et al., 2020b), as well as intercultural knowledge (Chu et al., 2019; Diego-Mantecón et al., 2021). There is no consensus on whether STE(A)M practices should combine two (or more) disciplines (Carmona et al., 2019; Maass et al., 2019a) or should integrate all (Martín-Páez et al., 2019; Toma & García-Carmona, 2021). Although STE(A)M is an emerging approach, it is already linked to various learning methodologies, project-based learning being the most common. PBL is a student-centred method in which students adopt an active role and teachers act as facilitators of the learning process. Thibaut et al. (2018) contemplated five PBL dimensions, as follows: content integration, problem-centred, inquiry-based, design-based, and cooperative learning.

Content integration implies combining knowledge and skills from STE(A)M disciplines, with one discipline playing a dominant role (Martín-Páez et al., 2019). Three approaches to content integration are usually described: multidisciplinary (Conradty & Bogner, 2019; Kim, 2016), interdisciplinary (Chaaban et al., 2021) and transdisciplinary (Herro & Quigley, 2017; Quigley et al., 2020b). The multidisciplinary approach entails learning content separately in each discipline but within a common theme (English, 2016; Gresnigt et al., 2014). The interdisciplinary approach juxtaposes content from at least two disciplines, establishing explicit connections (Gao et al., 2020). In the transdisciplinary approach “the curriculum transcends the individual disciplines” (Gresnigt et al., 2014, p. 52) and knowledge and skills are applied in real-world situations (English, 2016; Gresnigt et al., 2014). Apart from these three approaches, some authors considered the monodisciplinary one (Gao et al., 2020), which is not a STE(A)M integrated approach as it incorporates content from a single discipline (Toma & García-Carmona, 2021). The second dimension, problem-centred, implicates solving problems in authentic contexts (Conradty & Bogner, 2019; Margot & Kettler, 2019). These problems tend to be open-ended and ill-defined, encouraging creative solution pathways (Herro et al., 2019) and multiple answers (Diego-Mantecón et al., 2021). Inquiry-based learning seeks to promote processes such as questioning, hypothesizing, experimenting, and deducing conclusions (Pedaste et al., 2015; Thibaut et al., 2018). In the design-based dimension, engineering and technology are central (Li & Schoenfeld, 2019): technology is viewed as a tool to create and test artefacts (Akgun, 2013) and engineering is viewed as the context to apply mathematical and scientific content (Margot & Kettler, 2019). Design-based learning fosters problem solving and creativity, facilitating mathematical knowledge acquisition (Li & Schoenfeld, 2019), reasoning (English & King, 2019), and positive attitudes toward mathematics (Diego-Mantecón et al., 2019). The fourth dimension, collaborative learning, emphasizes teamwork—“students working together for a common purpose” (Chapman et al., 2010, p. 39). According to Chu et al. (2019), teamwork helps students to examine phenomena and to relate new knowledge to existing knowledge. It also provides opportunities for generating discussions, solving conflicts, and communicating openly (Chaaban et al., 2021).

To characterize STE(A)M-PBL from a mathematics perspective, Li and Schoenfeld (2019) propose the framework Teaching for Robust Understanding (TRU, Schoenfeld, 2014, 2018, cf.). This framework includes five dimensions, as follows: mathematics; formative assessment; equitable access; cognitive demand; and agency, ownership and identity. Schoenfeld (2018, p. 494) states that these dimensions “are necessary and sufficient to characterize the kinds of teaching that result in students being knowledgeable, flexible, and resourceful thinkers and problem solvers”. The mathematics dimension captures the extent to which this discipline is emphasised and how the connections among procedures, concepts, and contexts are addressed. The formative assessment and equitable access dimensions concern the extent to which intellectual environments are created for all students, and whether they are equally encouraged and supported to share their thinking, with a meaningful feedback loop. The cognitive demand dimension refers to whether the aforementioned environments generate interactions that lead to mathematical enlargement. Smith and Stein (1998) distinguish between lower- and higher-level cognitive demands. The former encompass ‘memorization’ and ‘procedures without connection’: memorization refers to tasks where previous facts, concepts, and processes are reproduced, and procedures without connection relate to activities where the application of the method is evident. The latter comprise ‘procedures with connection’ and ‘doing mathematics’. Procedures with connection cover tasks where methods are applied to develop deep mathematics understanding, whereas doing mathematics includes activities in which the nature of mathematical concepts, processes and relationships is explored. The enhancement of cognitive demands not only depends on project characteristics, but also on how mathematics is presented and executed (Li & Schoenfeld, 2019; Stein et al., 1996). The agency, ownership, and identity dimension explores the extent to which students are provided with formative opportunities that promote their confidence in mathematics, as well as positive attitudes toward this discipline. Positive attitudes and self-confidence are affective dimensions of learning related to high mathematics performance (Hemmings et al., 2011).

## STE(A)M-PBL design

Thibaut et al.’s (2018) dimensions of well-designed STE(A)M projects are not always adequately addressed, obstructing the creation of the formative mathematics environments described by Li and Schoenfeld (2019). Regarding content integration, Potari et al. (2016) suggest that many proposals do not offer natural overlap among disciplines as educators often struggle to integrate these. Carmona et al. (2019) identified that some projects contain fewer discipline-connections (multidisciplinary projects) than others (interdisciplinary or transdisciplinary projects). The issue however does not seem to reside just in the number of disciplines to integrate, but also in the way content is incorporated and in the role that disciplines play. Martín-Paéz et al. (2019) found that mathematics rarely plays a dominant role in many proposed experiences. Other authors go further, indicating that when mathematics emerges either it does not match curricular content or it relates to basic arithmetic (Diego-Mantecón et al., accepted; Lasa et al., 2020; Siverling et al., 2019). The results of Siverling et al.’s (2019) study, for instance, revealed that two of the seven analysed projects did not include content aligned with the intended mathematics standards in the USA.

Concerning problem-centred learning, research has shown that STE(A)M projects are hardly contextualised in real life (Potari et al., 2016; Quigley et al., 2020a). Most proposals have no meaning outside school, because of the difficulty of being set in real world contexts (Domènech-Casal et al., 2019). Potari et al. (2016) suggested that the ability to set a context relates to educators’ specialization, being more challenging for mathematics than for science teachers. Researchers point out similar difficulties in the design of ill-defined problems; for instance, modelling activities usually require either a unique path to attain the solution (Dogan, 2020) or the application of existing models through technology (Domènech-Casal, 2020). Difficulties in including the inquiry-based learning dimension have also been identified. Toma et al. (2017) detected, in an experimental study with pre-service primary teachers, that two thirds of the individuals struggled to meaningfully integrate the inquiry dimension; their proposals lacked empirical studies for analysing variables. Moraga et al. (2019) arrived at similar conclusions when examining the chemistry units proposed by pre-service high school teachers.

In relation to the design-based learning dimension, Gao et al.’s (2020) systematic analysis of about 40 articles revealed that educators tend to incorporate design processes in their projects. However, many of these projects do not necessarily promote mathematical and scientific concepts as a learning goal (Estapa & Tank, 2017). Diego-Mantecón et al. (accepted) and Lasa et al. (2020) pointed also out that projects with a design focus usually seek to illustrate the functioning of artefacts and hardly promote environments that facilitate the learning of mathematics. Regarding collaborative learning, researchers tend to concur that this dimension is frequently addressed in STE(A)M projects to stimulate divergent thinking abilities (Catarino et al., 2019) and to generate different approaches and solution strategies (Estapa & Tank, 2017).

## STE(A)M-PBL implementation

As far as these authors know, systematic analyses of STE(A)M-PBL instruction from a mathematics perspective have not been attempted. Insights about some dimensions of this approach were however reported. In a study with in-service teachers, Diego-Mantecón et al. (accepted) suggested that engineering-PBL instruction is rather shaped by teachers’ specialisation. In this vein, Herro and Quigley (2017) and Potari et al. (2016) indicated that the way teachers explain a concept is influenced by their academic degree and teaching experience; they for instance observed that conversions and functions are taught differently in science and mathematics lessons. In projects with emphasis on design, technology teachers tend to exploit engineering aspects, avoiding justifications from science and mathematics (Burghardt & Hacker, 2004; English, 2019). The tendency to pay attention to the artefact construction, and neglect other parts of the project, was also observed in Colombian high schools (Macías et al., 2020). Macías et al. claimed that the final product cannot be understood as the only objective and all disciplines should be similarly approached during implementation. To facilitate instruction endorsing formative assessment and high cognitive demand for all, Berardi and Corica (2021) and Quigley et al. (2020a) proposed involving students in real contexts meaningful to them. The mathematics to be applied in such contexts may be difficult but teachers could simplify it (Izaguirre et al., 2020; Macías et al., 2020). In a 2-year STEAM program, Diego-Mantecón et al. (2019) detected that projects emphasizing engineering and technological components helped low-average school mathematics achievers to develop a practical sense of the applicability of this discipline and positive beliefs about its learning.

There seems to be no agreement on the role teachers and students should adopt during the STE(A)M project implementation. Some authors suggest that projects should be led by students, especially during the inquiry phase, as they are more likely to promote processes such as examining, questioning, and hypothesising (Quigley et al., 2020a). Contrarily, others claim that teachers must guide the learning process because students are able to pose only those questions that emerge naturally in their minds (Berardi & Corica, 2021). When software like GeoGebra is required to approach a project, students need support and cannot be left freely in all steps of the practice (Blanco et al., 2019). Concerns arise also about how to deal with collaborative learning during the instruction (Nguyen et al., 2021). Sometimes teachers struggle to guide students in a meaningful way, as the latter may proceed freely eluding mathematics engagement (Diego-Mantecón et al., accepted). Chaaban et al. (2021) and Nguyen et al. (2021) stressed that many teachers abandon collaborative learning due to students’ resistance to change, and the fact that it generates low levels of effort in some students. To avoid this issue, Diego-Mantecón et al. (2021) proposed working in small groups under the KIKS (Kids Inspiring Kids for STEAM) format. The KIKS format promotes collaboration not only among the members of a single team, but also among other educational agents including national and international counterparts, teachers, families, and researchers.

## Research questions and methods

Although several STE(A)M-PBL projects are currently under way, there is little information about their implementation in the classroom and impact on school mathematics learning. To bring light to this matter, we address the following questions: How do novice teachers in STE(A)M-PBL implement this approach in their classroom to foster mathematics learning? and What PBL dimensions do they tend to emphasise? To answer these questions, we analysed 41 projects implemented by 11 in-service teachers. The research analysis is qualitative in nature although data were coded to categorise outcomes. The study does not aim to generalise results but to gain insights into how the STE(A)M-PBL instruction is articulated, from a mathematics learning perspective.

### Sampling selection and description

The 11 in-service teachers are Spanish and part of the Open STEAM professional-development program, at the University of Cantabria. This program comprises two iterative phases: the first provides teacher training, supervision and resources on STE(A)M-PBL; the second entails teachers’ project implementations in their classrooms. These two iterative phases of training and implementation began in September 2015, and were interrupted by the COVID-19 pandemic in 2020. In phase I, teachers received theoretical lessons on STE(A)M-PBL and attended workshops for reproducing projects. The workshops were organised in three sessions; the first (2–3 h) delivered information about the teaching approach and how it fits into the Spanish educational system. The other two sessions (2–3 h each) involved the execution of projects. Teachers arranged in groups of four worked collaboratively, experiencing the same difficulties their students would face in the classroom. Although the proposed initiatives integrated interdisciplinary content, the way it was incorporated and the number of disciplines involved varied across projects. The training phase was constantly refined throughout the program according to the necessities of the implementation phase.

Teachers were free to decide on the projects they would implement and the way of executing them. They selected projects from either the program repository (https://www.opensteamgroup.unican.es/) or their own harvest. The projects in the repository contain a description guide with information about the content, recommended age, and suitable number of participants; videos of classroom experiences are also included. Members of the Open STEAM Group assisted teachers during the implementations. Although they were unrestricted in many aspects of the instruction, teachers were encouraged to follow the KIKS format (Diego-Mantecón et al., 2021 cf.). This format establishes a well-defined elaboration process in which projects are introduced by a challenge: how can we get other students interested in STE(A)M disciplines? Teachers and students choose the project; once settled, students proceed to outline, sequence, and distribute tasks. All team members collaborate from the initial proposal to the conclusion, executing inquiry and design processes, as well as sharing information and reaching agreement. Students work in a non-native language, usually English, to motivate those from abroad. They must prepare a report and a video; the report describes the project, its development, and the results, emphasizing analytical aspects, while the video contains practical aspects of the artefacts’ construction and applicability. Students have also to present their work to different audiences in on-line or face-to-face encounters, nationally and internationally.

#### Teachers’ characteristics

Although initially 107 teachers from 36 schools began the program, only 11 are now actively participating in the Open STEAM community. The 11 teachers belong to eight educational centres from a region in the north of Spain. They teach mathematics, physics, chemistry, technology and biology regular lessons at different middle- and high-school levels. Some teachers instruct more than one discipline, depending on the school’s necessities. They have different backgrounds: five hold a mathematics degree, three are engineers, one is a physicist, one a biologist, and one a chemist. The mathematicians studied pure mathematics—learning mainly analysis, algebra, geometry, topology, and statistics—and received little or no training on physics or other subjects. The physicist studied a combination of physics and mathematics subjects focused primarily on algebra and analysis. The engineers, chemist, and biologist also studied mathematics subjects (e.g., analysis, geometry or statistics), in addition to their main areas. All teachers, apart from one, are over 40 years old and have more than 15 years of experience as educators, but not in STE(A)M-PBL. The 11 teachers (7 from state-subsidised schools and 4 from state schools) are motivated individuals, investing time outside school hours to prepare activities and support students. As part of the program, these teachers implemented STE(A)M projects in their classrooms with students aged 14–18. The teachers who left the program gave the following reasons: not having time; not finding benefit in STE(A)M education; not being supported by their schools; not considering that this approach fits into the curriculum; or not feeling confident in its implementation.

### Data collection and analysis

To analyse the 41 implementations, data were collected from the beginning to the end of the multi-year program, through direct observations, in classrooms and events, and by means of semi-structured interviews with the teachers. For the observations, we used rubrics that are summarized in Table 1. The rubrics were structured in relation to the 10 dimensions of Thibaut et al. (2018) and Schoenfeld (2014), the four types of projects described (mono-, multi-, inter-, and trans-disciplinary), and teachers’ specialisation (in- and out-of mathematics field). Thibaut et al.’s and Schoenfeld’s frameworks allowed the categorisation of the projects and the mathematical characterisation of the instruction, respectively. For each dimension we considered keywords from its conceptualisation in the present study. For example, to collect information about cognitive demand we considered keywords such as memorisation, and meaningful application of concepts (Table 1). The data from the rubrics was matched with the analyses of the projects elaborated by the students (analytical document and video), and the information extracted from teachers’ interviews. The interviews were executed before, during, and after project implementation for each dimension, refining these as the program proceeded. To collect data about the agency, ownership and identity dimension, we asked questions such as: ‘Do you consider your students modified their perception about the applicability of maths, and their confidence in maths?’ In addition, teachers were asked to report any significant increase in the marks obtained by the students in regular mathematics assessments.

**Table 1:** Rubric for analyzing STEAM-PBL implementation

Content integration	Problem	Inquiry	Design	Collaborative	Teacher
Monodisciplinary Multidisciplinary Interdisciplinary Transdisciplinary	Real/contextual Ill-defined …	Questioning Gathering/analysing data …	Models Designing solutions …	Working in groups Communicating …	In-field Out-of-field Collaboration

Maths content	Formative assessment	Equitable access	Cog. demand	Agency, ownership, and identity
Numbers Algebra … Matching curricular standards	Feedback loop Promoting thinking …	Poor participation Engagement …	Memorization Meaningful applications …	Maths applicability Self-confidence …

## Results

From the 41 projects, eight were identified as monodisciplinary, 0 multidisciplinary, 27 interdisciplinary, and six transdisciplinary (Table 2). Monodisciplinary projects were implemented at the beginning of the program by teachers initially lacking confidence in the integrated approach. These projects usually entailed science instruction, combining biology, chemistry, and/or physics (e.g., Chocolate Composition, Artificial Satellites) and led by out-of-field mathematics teachers who reproduced activities familiar to them. This instruction was characterised by the inquiry-based and collaborative learning dimensions, with no emphasis on mathematical content (Table 3).

**Table 2:** Projects emphasizing Thibaut et al.’s dimensions

	Content integration	Problem	Inquiry	Design	Collaborative
Monodisciplinary	8	1	8	0	8
Multidisciplinary	0	0	0	0	0
Interdisciplinary	27	22	23	13	27
Transdisciplinary	6	6	6	6	6

**Table 3:** Projects emphasizing mathematics TRU dimensions

	Maths content	Formative assessment	Equitable access	High Cog. demand	Agency, ownership, and identity
Monodisciplinary	0	0	0	0	0
Multidisciplinary	0	0	0	0	0
Interdisciplinary	22	22	22	15	15
Transdisciplinary	3	3	3	0	0

The 27 interdisciplinary projects corresponded to experiences in which teachers established explicit connections by juxtaposing content from at least two disciplines (e.g., Arches in Our City, Determining Geographical North). As Table 3 shows, 22 projects offered opportunities to work with mathematical content and provided formative environments with equitable access, 18 also promoted medium–high cognitive demand and skills in mathematics, as well as positive attitudes towards this discipline. Although transdisciplinary projects were encouraged, only six were implemented; these real-world experiences naturally connected disciplines and accounted for variables often simplified in regular lessons (e.g., Vertical Gardens, Floating Nest). Three transdisciplinary projects incorporated mathematical content and provided formative assessment and equitable access; however, none enhanced medium–high cognitive demand and student identity in mathematics (Table 3).

Below, we report how teachers approached the 22 interdisciplinary and three transdisciplinary projects emphasising mathematics. Twelve interdisciplinary lessons were executed by in-field teachers, five by out-of-field, and five by mixed teams of both kinds of teachers (Table 4). The instruction of in-field teachers was characterised by problem-centred (9), inquiry-based (11) and collaborative learning (12), while out-of-field teachers focused on problem-centred (5), design-based (4), and collaborative learning (5). The mixed collaborations involved all Thibaut’s dimensions, apart from design-based learning. The three transdisciplinary projects encompassed all dimensions (Table 4).

**Table 4:** Projects involving maths by teacher specialization

	Content integration			Problem			Inquiry			Design			Collaborative		
	In	Out	Coll	In	Out	Coll	In	Out	Coll	In	Out	Coll	In	Out	Coll
Interdisciplinary	12	5	5	9	5	5	11	3	5	4	4		12	5	5
Transdisciplinary		1	2*		1	2*		1	2*		1	2*		1	2*

	Maths content			Formative assessment			Equitable access			High Cog. demand			Agency, ownership, and identity		
	In	Out	Coll	In	Out	Coll	In	Out	Coll	In	Out	Coll	In	Out	Coll
Interdisciplinary	12	5	5	12	5	5	12	5	5	9	1	5	9	1	5
Transdisciplinary		1	2*		1	2*		1	2*						

*Projects implemented through an out-of-field mathematics teacher collaboration

Content related to geometry appeared in 14 of the 25 projects, numbers and statistics in 11, functions and algebra in 7, and probability in 1 (Table 5). Geometry emerged in projects led by in- and out-of-field teachers because of its presence in nature and usage for representing designs (e.g., Golden Number, Star Wars Robot). Functions were found mainly in the collaboration of mathematics and science teachers for modelling data to explain real-life phenomena (e.g., Modelling Objects in Motion). In all projects involving mathematics, formative assessment and equitable access were promoted. Regardless of specialisation, teachers reproduced formative mathematical environments for all providing feedback and interaction. Through the KIKS format, students were supported to share their thinking, with a meaningful feedback loop for learning adjustment. During project elaboration and presentation in events, all students had the opportunity to engage with mathematical content and practices so that every student could profit from it.

**Table 5:** Teacher specialization, maths content, cognitive demand, and identity

Content/formative/access	In	Out	Coll	Cognitive demand	In	Out	Coll	Identity	In	Out	Coll
Geom.	11	3		Memorization	3			Maths applicability into different situations	8	1	5
Functions	1	1	4	App. w/o connection		5	2*	Maths interrelation with other subjects	8	1	5
Statistics	2	2	1	App. with connection	6		3	Maths confidence	8	1	5
Numbers	3	1	2*	Doing maths	3	1	2	Maths value	9	1	5
Algebra	1										
Probability	1										

*Projects implemented through an out-of-field mathematics teacher collaboration

Although learning opportunities were offered in all projects, the way teachers delivered content varied. Three projects of the in-field teachers promoted low cognitive demand, such as the identification and memorisation of mathematics concepts and facts; these were golden number-related tasks implemented at the beginning of the program as part of the first teachers’ experimentations. In-field teachers also built in environments requiring cognitive demand matching curricular standards; nine projects promoted medium–high demand through the application of concepts and/or procedures with a specific meaning in the projects, and five encouraged high demand (doing mathematics) through the analysis, generalisation and justification of mathematics results for interpreting project outcomes (Table 5). For example, in the Arches in Our City project tasks focused on mathematically analysing artistic and historic creations to reproduce a semi-circular arch, representative of the students’ city, using geometry. In projects with strong design-based learning (e.g., Star Wars Robot, Hydrobot), led by engineering teachers, mathematics was rarely exploited. Content endorsed low cognitive demand concerning basic geometry (e.g., identifying/drawing shapes corresponding with the artefacts’ components) and basic arithmetic (e.g., calculating material cost). Similarly, science teachers instructed projects centred on inquiry-based learning (e.g., Microorganisms on Everyday Objects, Floating Nest). The mathematics in these lessons was also comprised under students’ curricular standards, involving frequencies to represent data or budget calculation. That is, out-of-field teachers worked the mathematics naturally emerging from tasks (normally basic) or the ones required by the KIKS format to disseminate results; only one project promoted high demand in mathematics. In-field teachers’ tendency to avoid design for exploiting mathematics was confirmed when one of these teachers was requested to implement the following project: Constructing a Robot for Solving Rubik’s Cube. Unlike the out-of-field teacher, the mathematics teacher advanced the robot design by directly assembling Lego pieces. He then explained intensely the concept of algorithm by establishing a relationship between the robot’s movements and arrangement of students’ hands when solving the cube. The mathematics teacher did not just stimulate searching for an algorithm to be applied but also emphasised understanding its meaning; he challenged the students to calculate the possible number of movements fostering reasoning.

The three transdisciplinary projects incorporating mathematical content, formative assessment and equitable access did not promote high cognitive demand nor encourage positive attitudes toward mathematics. These were real-world projects naturally connecting disciplines and considering variables simplified in regular teaching (e.g., A drop of Life, Floating Nest). These experiences were of complex execution, demanding in- and out-side time-consuming classroom activities where teachers applied knowledge from disciplines distinct to their specialisation. For the Floating Nest project, aiming to protect the great crested grebe (an aquatic bird) at risk of extinction for not having natural surfaces of reproduction, participants bought materials for constructing nests, tested the viability of prototypes through experiments, and got governmental authorization for locating platforms in the lake. Although this project naturally integrated knowledge and skills from the four STEM disciplines, the mathematics required was basic, related to school computations and data representation. The interviews revealed that in-field teachers avoided these projects because they were time consuming and involved considerable casuistry. Teacher 10 (male mathematician aged 52) stressed “These authentic experiences are exciting but require much time and close collaboration for dealing with rather sparse maths”. These were lessons undertaken by science or engineering specialised teachers who simplified mathematics to the detriment of design. They were teachers from state-subsidised schools who reported that they were better supported by their centres than their peers from state schools. All teachers agreed that successful transdisciplinary implementations require rescheduling subject timetables and establishing close collaboration of teachers. During the program we observed progressive cooperation among the educators due to continuous interactions in events. Five mixed-collaborations (in- and out-of-field teachers) generated interdisciplinary settings promoting medium–high cognitive demand, and two out-of-field collaborations raised transdisciplinary experiences endorsing medium–low cognitive demand (Table 5).

Despite these formative opportunities, the teachers reported that student marks in regular mathematics assessments did not improve. This is plausible because, as shown above, mathematical content was not present in most projects, was oversimplified, or below curricular standards. Regardless of this fact, the 11 teachers agreed through interviews that the knowledge acquired during the projects was longer maintained than that acquired in regular lessons. Teachers conveyed that this aspect was influenced by PBL and KIKS format features. Teacher 4 (male mathematician aged 55) stated “Students can remember for longer the ideas and concepts applied in STEM activities, because they are really involved in the resolution processes”. Reaching contextualised solutions, in collaboration with their counterparts, and presenting these in events, enabled also profound mathematical engagement. Teacher 11 (male mathematician aged 51) indicated “Working in an international setting allowed me and my students to get useful feedback from others and improve the projects”.

Although the projects incorporating mathematics facilitated equitable access, students did not always benefit from it. Some students were unwilling to invest effort and time as their goal was to obtain high marks in national examinations, or they were not interested in school learning. Teachers reported, and we observed, that students engaged differently in distinct parts of the project irrespective of the opportunities provided: some individuals worked on practical tasks as hands-on activities and refused to contribute in analytic processes or in disseminating results. Consequently, teachers had reservations about collaborative learning, despite recognising its potential to generate rich mathematical environments. As they confirmed, some students tended to assume roles linked to their skills or knowledge, not promoting aspects in which they lacked confidence. This aspect connects with another of the teachers’ concerns—the evaluation process. Teachers expressed the difficulty of assessing students in an equitable manner, agreeing that collective evaluations are unfair and unrealistic. Teacher 7 (male mathematician aged 64) asserted “It’s difficult to evaluate students as a group because marks should truly correspond with the standards attained by each student”. The interdisciplinary projects helped to gain a realistic perception of the mathematics applicability in a variety of contexts and in relation to other subjects. According to teachers, students became aware of the applicability of mathematical concepts and procedures learned at school in at least 8 projects (Table 5). Teacher 10 (male mathematician aged 52) stated “My students were unaware of the importance of maths for solving problems in context until they started elaborating these projects”. For example, in the project Design and Construction of an Astrolabe, students used mathematical tests to verify the astrolabe accuracy until they calibrated it. Similarly, in the project Modelling Objects in Motion, teachers provided opportunities to explore the properties of functions not often observed in mathematics lessons. Teacher 11 (male mathematician aged 51) expressed “My students used maths to represent the trajectory of objects in motion to then verify results using technology”. These experiences (at least 15) made students value mathematics and become more confident in this subject (Table 5). Teacher 4 (male mathematician aged 55) pointed out “When in my lessons I work maths without a context, students often ask the reasons for learning it”.

## Discussion

The results showed that across a STE(A)M-PBL professional multi-year program, 11 in- and out-of-field teachers implemented 41 projects, with 25 incorporating some kind of mathematical content. In these 25 projects, teachers provided formative mathematical environments with equitable access, with 15 promoting medium–high cognitive demand in mathematics, and positive attitudes towards this discipline. This study demonstrates that, with the necessary time, teachers can progress from implementing monodisciplinary to interdisciplinary projects, and even transdisciplinary. Some teachers started by introducing monodisciplinary experiences, which do not integrate disciplines and thus are not STE(A)M projects, as characterized by authors such as Martín-Páez et al. (2019) and Toma and García-Carmona (2021). The transition towards integrated teaching was partially due to the continuous teacher interaction within a supportive group. These findings concur with those of Potari et al. (2016), who reported that changes towards integrated instruction relate to a high willingness to improve and the sense of belonging to a successful professional community. Our study revealed that teachers needed several implementations and a feedback loop to move towards a more integrated approach, as suggested by Al Salami et al. (2017). Some teachers initially provided interdisciplinary experiences promoting medium–low cognitive demand in mathematics, but later provided environments for medium–high demand. Real-world experiences naturally connecting disciplines and taking into account many contextual variables (transdisciplinary projects) were even undertaken, although these still promoted only medium–low cognitive demand. This observation is in line with the results of Domènech-Casal et al. (2019), who reported that many STEM proposals do not make sense outside school or are barely implicated in everyday situations.

In-field mathematics teachers, as well as the mixed-collaborations, promoted medium–high cognitive demand in 14 interdisciplinary projects, using a variety of mathematical content. These were often projects centred on problem, inquiry, and collaborative learning. In-field teachers tended to simplify design processes and encourage mathematics through tasks where concepts and procedures were meaningfully applied, working out the cognition requested in typical textbooks; this result is a finding also reported by Vásquez et al. (2019). Out-of-field teachers endorsed mainly medium–low cognitive demand tasks in interdisciplinary projects with a focus on problem, design, and collaborative learning. The mathematics in these projects involved identifying and drawing geometrical shapes, and the application of basic procedures, very much related to project design. This result resonates with those of Burghardt and Hacker (2004), English (2016), Lasa et al. (2020), and Macías et al. (2020), who exposed the difficulties of addressing mathematical content in proposals centred on design. The transdisciplinary instruction of out-of-field teachers did not facilitate exploiting mathematics and thus promoting cognitive demand. The mathematics emerging here involved calculations and data representation to deliver results. Despite the common thought that mathematics is universally present, it did not emerge naturally in these projects. The above result suggest that interdisciplinary projects facilitate exploiting school mathematics, while transdisciplinary ones encompass real-life situations where mathematics occurs in a less explicit manner. The latter coincides with Berardi and Corica’s (2021) statement that in STEM activities mathematics does not emerge naturally, and effort is necessary for it to arise.

Despite formative assessment, and the promotion of cognitive demand in several interdisciplinary projects, the teachers did not identify changes in students’ mathematics marks in ordinary exams. It is noteworthy that in a similar context, Han et al., (2015, 2016) found significant differences in achievement. Reporting in projects with an engineering design focus, Diego-Mantecón et al. (2019) did not find changes in mathematics scores. The teachers in this study testified that knowledge acquired from STE(A)M-PBL is maintained longer because the learning processes were central for the students. Due to the PBL characteristics and the KIKS format, they worked for months elaborating projects and preparing presentations for different audiences, which promoted longer retention of the knowledge and skills involved. This experience also modified some of their perceptions of mathematics; in projects of the in-field mathematics teachers, students became aware of the applicability of this subject to other areas and contexts, feeling more confident and valuing it more. These findings also concur with those reported by Diego-Mantecón et al. (2019) with low achievers. Despite the teachers facilitating equitable mathematical environments for all students, the latter did not equally engage in the learning process. This result confirms that even when instruction is well executed, regardless of the method, learning requires student effort and commitment. In this light, Chaaban et al. (2021) and Nguyen et al. (2021) stated that numerous teachers abandon innovative educational practices due to students’ resistance to cooperation. The collaborative learning dimension of the STE(A)M-PBL was contradictorily valued by the teachers, reporting this aspect of the instruction to be beneficial for some students and not for others. All teachers agreed, however, on the difficulty of assessing individuals in groups, suggesting that there is no equitable manner to provide grades; a finding already highlighted by Margot and Kettler (2019). In this regard, Thibaut et al. (2018) argued that it is still unclear how STEAM projects should be assessed. Cuéllar and Alonso (2010) suggested that assessment should not just consider students’ solutions, but other aspects such as the interactions facilitating knowledge construction.

## Conclusions and implications

This study shows that, under a continuous professional-development program, teachers progressed towards the implementation of the STE(A)M-PBL approach. However, changes occurred slowly over time. In about 4 years of work, they accomplished 25 projects with emphasis on mathematics (out of 41 attempts), and only 22 of these projects turned out to promote some kind of cognitive demand, and the enhancement of student mathematical identity. Out-of-field teachers faced difficulties in naturally exploiting mathematics, but they moved to a more integrated approach than in-field mathematics teachers, in which they completed transdisciplinary projects. In-field teachers achieved more interdisciplinary experiences, creating formative mathematical environments to endorse high demand and positive perceptions about this discipline (14).

These authors believe that the 11 teachers who remained in the program can still progress in their instruction in future years, but we are unsure whether transdisciplinary projects are suitable for exploiting school mathematics in an effective way. The number of contextual variables that these real projects involve is large, and these may not be worth considering if the aim is attaining the intended mathematics curriculum. Certainly, further research would be necessary to confirm or reject this assumption. Interdisciplinary projects, also time consuming, seem to be an option for teachers to introduce school mathematics within an STE(A)M integrated learning. The contextualised (not real) characteristic of these projects, however, makes it difficult to prepare our students for the necessities of real life as contexts are usually simplified. At this stage, we must consider again curricular objectives: Should we train our students in mathematics to provide them with a strong mathematics foundation? or Should we prepare them for solving real-life situations that involve mathematics?

We believe that at school level students require consolidating mathematical groundwork necessary for the learning of related disciplines, and consequently for accessing STEM careers or professions. We suggest combining traditional teaching with integrated content approaches (interdisciplinary or transdisciplinary), and evaluating how both coexist. Students should be able to apply mathematics in context, but also should know the mathematical rules and procedures needed to meet this first requirement. Incorporating in the school scheme a STE(A)M subject, instructed by at least two teachers, may be a starting point, as radical changes should not be introduced in education.
